# Long-Lasting Sound-Evoked Afterdischarge in the Auditory Midbrain

**DOI:** 10.1038/srep20757

**Published:** 2016-02-12

**Authors:** Munenori Ono, Deborah C. Bishop, Douglas L. Oliver

**Affiliations:** 1 Department of Neuroscience, University of Connecticut Health Center, Farmington, CT, USA 06030-3401; 2 Department of Physiology, Kanazawa Medical University, Ishikawa 920-0293, Japan

## Abstract

Different forms of plasticity are known to play a critical role in the processing of information about sound. Here, we report a novel neural plastic response in the inferior colliculus, an auditory center in the midbrain of the auditory pathway. A vigorous, long-lasting sound-evoked afterdischarge (LSA) is seen in a subpopulation of both glutamatergic and GABAergic neurons in the central nucleus of the inferior colliculus of normal hearing mice. These neurons were identified with single unit recordings and optogenetics *in vivo*. The LSA can continue for up to several minutes after the offset of the sound. LSA is induced by long-lasting, or repetitive short-duration, innocuous sounds. Neurons with LSA showed less adaptation than the neurons without LSA. The mechanisms that cause this neural behavior are unknown but may be a function of intrinsic mechanisms or the microcircuitry of the inferior colliculus. Since LSA produces long-lasting firing in the absence of sound, it may be relevant to temporary or chronic tinnitus or to some other aftereffect of long-duration sound.

Neural plasticity is one of the central issues in the brain^1^. Neural plasticity has different forms that underlie the various brain functions such as learning, feature extraction, etc. In the central auditory system, different forms of neural plasticity are known to play a critical role in the computation of auditory information. For example, in the auditory brainstem short term synaptic depression has been reported to improve coincidence detection required for sound localization^2^. In the auditory cortex, long term synaptic potentiation was found to reduce the response variability to improve sound perception^3^.

The inferior colliculus is a critical auditory interface between the brainstem and the forebrain. As the major nexus for inputs ascending from the lower brainstem, IC neurons integrate information about sound and transmit it to the higher centers of the auditory system^4^. The IC is not a mere relay, but is known to have top-down^5^,^6^ and bottom-up^7^,^8^ plastic mechanisms to modify the neuronal receptive field. The IC is unusual since it contains both glutamatergic and GABAergic neurons that project to the medial geniculate in the thalamus^9^,^10^,^11^. However, little is known about plasticity in the function of specific neuron types in the IC.

Here, we report a novel type of plastic neural activity in a subpopulation of neurons in the IC. These neurons showed a distinct long-lasting sound-evoked afterdischarge (LSA) that was an increase in firing that lasted from seconds to minutes after the sound termination. During the sound presentation, neurons with LSA had less accommodation than cell types without. Both GABAergic and glutamatergic neurons, distinguished optogenetically *in vivo*, had LSA. These findings suggest that the mechanism to generate LSA might counteract the neural accommodation due to synaptic depression or Ca^++^-activated K^+^ channel conductances that decrease firing rates during long-duration sound stimulation.

Normally, sound stimulation creates neural activity, and this leads to the perception of sound. Since activity in the IC signals the auditory forebrain that a sound is present, this long-lasting afterdischarge might signal that a sound is present when there is none and lead to a phantom sound perception.

## Results

**Figure 1:**
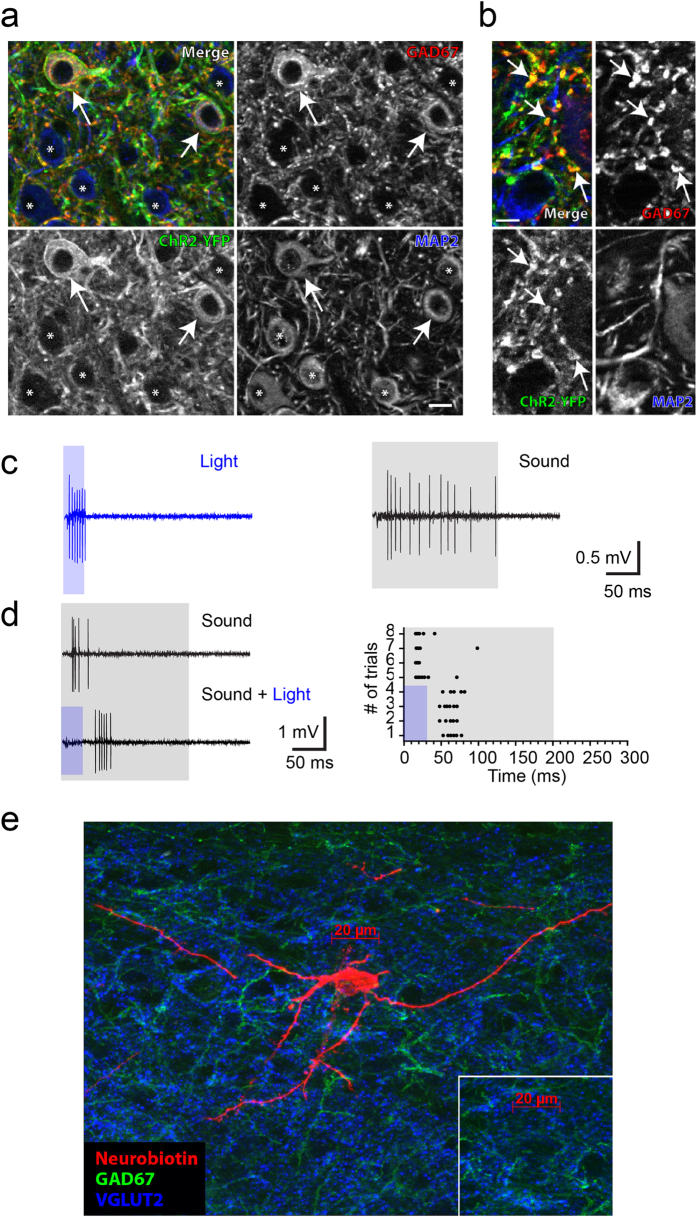
GABAergic and nonGABAergic neurons in IC were distinguished *in vivo*. (**a**) GAD67, ChR2-YFP, and MAP2 were immunohistochemically stained. Arrows show the neurons in which GAD67 and ChR2-YFP are colocalized. Asterisks indicate the non-GABAergic neurons with only MAP2. (**b**) GAD67 and ChR2-YFP are colocalized in axonal terminals. Scale = 10 μm. (**c**) A neuron activated by light. Left, the spike responses to light (30 ms, 50 mW). Right, response to 200 ms white noise (80 dB). Gray box indicates the sound presentation here and in subsequent figures. (**d**) A neuron suppressed by light. Left upper, the response to 200 ms white noise (80 dB). Left lower, the white noise with light (30 ms, 50 mW). Blue box indicates light presentation. The right panel is the raster plot of the response to noise and light. (**e**) An image of a juxtacellularly stained neuron that was suppressed by light. Note that it is GAD67 (green signal, a marker for GABA) negative. All the stained neurons with light suppression were GAD67 negative (n = 5).

VGAT-ChR2 (H134R)-EYFP mice^12^ with normal hearing were used to distinguish GABAergic from glutamatergic neurons *in vivo*. In this mouse, GABAergic and glycinergic neurons specifically express the Channelrhodopsin-2 (ChR2) in all parts of the neuron (Fig. 1a,b). When a light stimulus was delivered to the IC from the brain surface, it evoked spikes in some neurons in extracellular recordings (Fig. 1c, left panel blue). In contrast, light suppressed firing in response to sound in other neurons presumably due to the light evoked release of inhibitory transmitters from presynaptic axonal terminals (Fig. 1d, lower panel, black). The inhibitory neurons in the mammalian IC are exclusively GABAergic neurons, while glutamatergic excitatory neurons make up the majority of the neural population^13^. Thus, we identified the IC neurons activated by light as GABAergic, while we identified those whose response to sound was suppressed as glutamatergic neurons. The specific expression of ChR2 in inhibitory neurons ensures that the light activated neurons were GABAergic. However, it is possible that some GABAergic neurons are so heavily innervated by inhibitory inputs that light evoked inhibitory transmitter release suppresses their light-evoked firing as well as their response to sound. Thus, we used juxtacellular labeling to stain five light-suppressed neurons and determine if they expressed GAD67, a marker for GABA neurons (Fig. 1e). These light-suppressed neurons lacked GAD67 (Fig. 1e) consistent with the notion that that light-suppressed neurons are glutamatergic neurons (n = 5). We also juxtacellularly stained one light activated neuron, which was GAD67 positive (data not shown).

**Figure 2:**
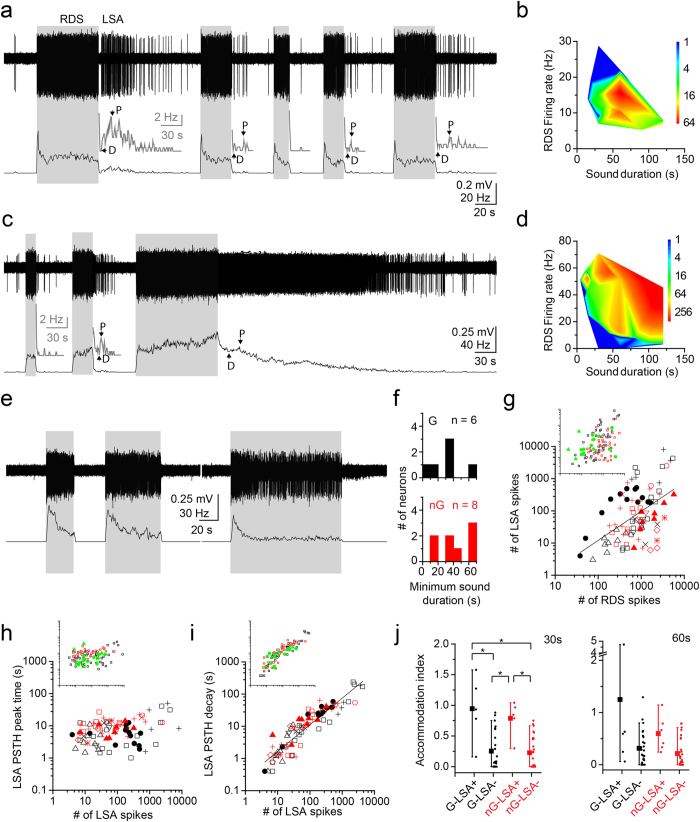
Both GABAergic and nonGABAergic neurons in IC had LSA. (**a,c**) The response of neurons with LSA to sound. (**a**) A nonGABAergic neuron. (**c**) A GABAergic neuron. Upper trace is the voltage spike trace of the response and the lower trace is its PSTH. The gray traces are magnified PSTHs. The arrows indicate the dip (D) and peak (P) of the PSTHs. The Gray boxes indicate the sound presentation. The sound intensity was 70dB in (**a)** and 80 dB in (**c)**. (**b,d**) Pseudocolor map shows the number of spikes within 30 s after the sound termination as RDS firing rate and sound duration were varied. (**b**,**d)** are the responses from the neuron in (**a**,**c**), respectively. (**e**) The response of a GABAergic neuron without LSA. The sound intensity was 70 dB. (**f**) Histograms of the minimum sound duration that evoked LSA in each neuron. (**g**) The number of LSA spikes was plotted against the number of RDS spikes. Different symbols indicate the responses from different neurons. Black and red symbols indicate the responses from GABAergic and nonGABAergic neurons, respectively, in this and subsequent figures. In the inset, the responses from CBA/J mice are indicated by green filled squares, circles, and triangles. (**h,i**) The peak time (**h**) and decay (**i**) of LSA PSTH are plotted against the number of LSA spikes. In the insets, the responses from CBA/J mice are indicated by green filled squares, circles, and triangles. (**j**) The comparison of the accommodation of RDS to 30 s (left) and 60 s (right) sound between the LSA+ and LSA− neurons. The larger index indicates less adaptive response in RDS. Black and red boxplots and dots indicate the response of GABAergic neurons and nonGABAergic neurons, respectively.

**Figure 3:**
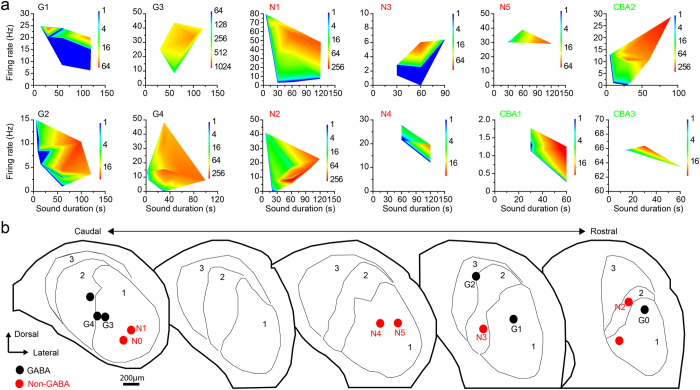
An overview of the responses of LSA+ neurons. (**a**) Pseudocolor maps of the number of spikes within 20 s after sound termination as shown in Fig. 2b,d. (G1-G4) GABAergic neurons. (N1-N5) nonGABAergic neurons. (CBA1-CBA3) the recordings from CBA/J mice. Pseudocolor maps were made when we successfully recorded the responses to more than three test sounds. The maps shown in Fig. 2 are excluded here. (**b**) The distribution of the LSA+ neurons in IC in VGAT-ChR2 mice. Black and red circles indicate GABAergic and nonGABAergic neurons, respectively. The location of each neuron was plotted on the schematic sections. Thin lines indicate the boundary of central nucleus of IC (ICC). ICC was separated in three different regions by the ratio of GABAergic and glycinergic terminals. In region 1, glycinergic terminals dominate. In region 2, there are relatively more GABAergic terminals than glycinergic terminals. In the most dorsal region (3) GABAergic terminals predominate although a few glycinergic terminals are still present. The numbers beside the circles correspond to the numbers in Fig. 3a. G0 and N0 correspond to the cells in Fig. 2d,b, respectively.

We found that 20% of glutamatergic (8/40) and 17% of GABAergic (6/36) neurons in the VGAT-ChR2 mice exhibited a long-lasting sound-evoked afterdischarge (LSA) and continued to fire after the sound terminated (Fig. 2a,c). The characteristic frequency (CF) and the threshold of the neurons with LSA were not significantly different from the neurons without LSA (Table 1; CF, p = 0.12; Threshold, p = 0.32; Kruskal-Wallis test). We tested 36 GABAergic neurons and 40 presumed glutamatergic neurons for LSA by using long duration, 30–60 s, continuous one-octave noise (see METHODS). All neurons were tested at 60 dB SPL. Time permitting, we also tested sound levels ranging from 30–90 dB SPL and durations ranging from 4–120 s. The LSA was stronger when the response during sound (RDS) was robust and the sound duration was long (Figs 2a–d and 3a). The minimum sound duration required to induce LSA was ~30 s (GABA, 28.3 ± 7.6 s, n = 6; nonGABA, 37.5 ± 7.5 s, n = 8, p = 0.42, Wilcoxon signed-rank test, Fig. 2f). In five GABAergic and five nonGABAergic neurons, we checked the minimal intensity required to evoke LSA using either a 30 or 60 s sound. The minimum intensity was 52.0 ± 6.6 dB (n = 5, range 30–70 dB) and 63.0 ± 3.0 dB (n = 5, range 60–75 dB) for GABAergic and nonGABAergic neurons, respectively, and not significantly different (p = 0.18, Wilcoxon signed-rank test). These levels were 20.0 ± 5.2 dB and 22.0 ± 6.8 dB above the threshold for GABAergic and nonGABAergic neurons, respectively (p = 0.75, Wilcoxon signed-rank test; Table 1). The number of spikes in the LSA and RDS was positively correlated (Fig. 2g, r = 0.51, p < 0.01, two-tailed Student’s *t* test). Six neurons were tested for LSA with both continuous and AM noise. Most showed LSA to both stimuli (2/3 GABA; 2/3 nonGABA). One nonGABAergic neuron had LSA to AM sound but not to unmodulated sound. Neurons exhibiting LSA were restricted to the central nucleus of IC (ICC) in the VGAT-ChR2 mice (Fig. 3b). Most LSA neurons were in area 1 (9 of 12 neurons) in the ventrolateral part of ICC, a region shown recently to have high concentrations of glycinergic axonal inputs^14^ (area 1, Fig. 3b). The remaining three neurons were in the adjacent area of ICC where GABAergic terminals are more prevalent than glycinergic terminals^14^ (area 2, Fig. 3b).

The time course of LSA was from seconds to minutes (Fig. 2a,c,h,i). At sound offset, there was a rapid dip in the firing rate (D in Fig. 2a,c) followed by a build-up to a peak firing rate (P in Fig. 2a,c) that occurred 1.0–50.1 s after sound termination. The GABAergic neurons had shorter peak times than the nonGABA neurons (GABA, 8.5 ± 1.5 s, n = 47; nonGABA, 10.6 ± 0.9 s, n = 40, p <0.01, Wilcoxon signed-rank test, Fig. 2h), but there was little correlation between peak times and number of LSA spikes (r = 0.29, p < 0.05, two-tailed Student’s *t* test, Fig. 2h). The duration of the decay of the LSA was strongly correlated with number of LSA spikes (r = 0.92, p < 0.001, two-tailed Student’s *t* test, Fig. 2i). The decays ranged from 0.4–235.6 s (GABA, 31.7 ± 8.1 s, n = 46; nonGABA, 16.9 ± 2.9 s, n = 39, p = 0.77, Wilcoxon signed-rank test, Fig. 2i).

To determine if LSA was due to the ChR2 transgenic strain, we tested CBA/J mice, a wild-type strain used in auditory research^15^. Recordings in CBA/J mice were made under urethane anesthesia to rule out ketamine, and its specific effects on NMDA receptors^16^, as a cause of LSA. In CBA/J mice, 15% (3/20) of IC neurons sampled showed an afterdischarge response that was essentially the same as that seen in the other conditions (Fig. 2g–i, insets, Fig. 3a). The minimum sound duration to evoke LSA was 23.3 ± 6.7 s (n = 3), and the minimum sound intensity was measured in two neurons (30 dB and 60 dB). The peak and decay time of LSA in CBA/J mice were 7.3 ± 2.2 s (1.4–38.2 s, Fig. 2h) and 10.0 ± 1.8 s (1.2–23.0 s, Fig. 2i), respectively. Thus, the LSA was not due to the use of a transgenic mouse with ChR2 or the use of ketamine anesthesia.

Neurons with LSA had less adaptation during sound than neurons without LSA (Fig. 2a,c,e,j). Some LSA neurons had increased firing during sound (buildup pattern, Fig. 2c) not seen in other neurons. We calculated an accommodation index (AI, ratio of firing rate of the last 2 s and the first 2 s of the RDS. Note, a larger value means less adaptation) to compare adaptation during RDS (Fig. 2j). In response to 30 s sound, both GABAergic and nonGABA neurons with LSA had more sustained RDS than neurons without LSA (LSA+ GABA AI = 1.01 ± 0.36, n = 5; LSA+ nonGABA AI = 0.82 ± 0.18, n = 5; LSA− GABA AI = 0.22 ± 0.06, n = 24; LSA− nonGABA AI = 0.22 ± 0.06, n = 26, p < 0.05, Wilcoxon signed-rank test, Bonferroni correction, Fig. 2j). A higher AI was also seen in response to 60 s sound in neurons with LSA, but it was not significantly different. (Fig. 2j; LSA+ GABA AI = 0.88 ± 0.49, n = 5; LSA+ nonGABA AI = 0.67 ± 0.24, n = 6; LSA− GABA AI = 0.39 ± 0.07, n = 27; LSA− nonGABA AI = 0.21 ± 0.05, n = 29). These results suggest that the mechanism to generate LSA is evoked during the sound presentation and might promote sustained responses to very long sounds.

**Figure 4:**
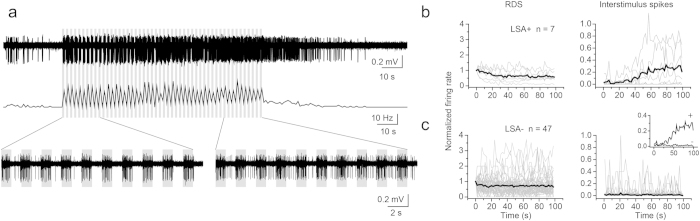
Discontinuous sound evoked LSA. (**a**) The response of a GABAergic neuron to sound with 50% duty cycle of 1 s (50 times repetition). Upper trace is the voltage spike trace of the response and the middle trace is its PSTH. The lower traces are magnified voltage traces. (**b,c**) The plots of RDS (left) and interstimulus spikes (right) against time. (**b**) LSA+. (**c**) LSA−.

Discontinuous sound could also evoke a LSA (Fig. 4a). We used 1 s narrowband noise bursts presented every 2 s in all neurons tested where LSA was first shown with continuous sound (50 repetitions, GABA, n = 5, nonGABA, n = 3). Interestingly, there was a gradual increase in interstimulus spikes (Fig. 4a,b) not seen in neurons lacking LSA (Fig. 4c). The interstimulus afterdischarge spikes began after 30 s of stimulation on average and continued until the termination of the 100 s train of noise bursts.

## Discussion

The present results suggest that a subset of neurons in the auditory midbrain is under the influence of a powerful but unknown phenomenon that potentiates continued firing after the offset of a sound stimulus. IC neurons might be expected to adapt and stop firing during long duration sound stimulation due to the depression of excitatory synaptic inputs which was seen in the majority of IC neurons^17^,^18^,^19^. However, the neurons with LSA showed less accommodation than non-LSA neurons, and consequently, their rate of firing during the sound stimulus was more sustained. This result suggests a mechanism in these IC neurons that may compensates for any synaptic depression and help the IC neurons to fire persistently to long sound. However, the LSA phenomenon is unlike long term depression or potentiation^20^ seen previously in IC neurons since it is not stimulus bound. It is also known that a subset of IC neurons has Ca^2+^-activated potassium conductances that are known to favor neural accommodation. Neurons with LSA are not likely to be the adapting or transient IC neurons with SK and BK currents, respectively, but instead the LSA neurons are more likely to be the sustained-regular or buildup-pauser neurons seen in previous *in vitro* studies^21^.

One possible mechanism underlying LSA is slow afterdepolarization (sADP)^22^,^23^,^24^. The size of sADP is proportional to the duration of the depolarization *in vitro*^22^, consistent with our result that LSA was well correlated with RDS (Fig. 2g). Previous *in vitro* studies of IC in rat have shown plateau potentials evoked by brief synaptic stimulation; moreover, the sustained-rebound and pause-build neurons have a depolarization that extends beyond the duration of the synaptic inputs^25^. Neuromodulators are relevant to sADP^22^,^24^,^26^,^27^, and the receptors for modulators such as dopamine^28^,^29^, serotonin^30^,^31^, acetylcholine^32^,^33^ and metabotropic glutamate (mGluR)^34^,^35^ are found in the IC central nucleus where LSA is seen. Previous studies have shown that sADP could be due to the activation of TRP channels^26^,^36^,^37^ and/or the inhibition of G-protein coupled inwardly rectifying K^+^ (GIRK) channels^26^. In the IC, TRPC1, GIRK1, and GIRK3 were reported to be expressed in neurons^34^,^38^. One recent study^34^ showed that both GABAergic and nonGABAergic neurons expressed TRPC1 channels and many of them co-expressed the group 1mGluR. It is possible that these channels shape sADP to generate LSA in IC.

A second possible mechanism underlying LSA is the recruitment of local microcircuits within the ICC. The primary disc-shaped neurons in ICC make extensive local axon collaterals within the fibrodendritic laminae that define the architecture of the nucleus^39^,^40^,^41^. Recently, glutamatergic ICC neurons were shown to make extensive synaptic inputs on neurons within the same layer^42^, and GABAergic neuron in IC may do so as well. Consequently, the ascending excitatory inputs to an ICC layer may stimulate multiple polysynaptic excitatory inputs within the same layer. The study of the ICC in the brain slice with voltage sensitive dye supports this view since it shows that localized responses to low-frequency stimulus trains spread when the stimulus frequency increases. This suggests the recruitment of silent microcircuits^43^. In the hippocampus, afterdischarges are seen as the rhythmic bouts of synchronized network activity in the clonic phase of seizure activity. These afterdischarges in CA3 pyramidal neurons require GABAergic synaptic transmission that becomes excitatory due to a transient collapse in the Cl^−^ reversal potential^44^. Thus, prolonged activity in IC local microcircuits may cause a shift in the Cl^−^ reversal potential of IC neurons and make normally inhibitory GABAergic synapses depolarize the neuron. This suggests that the long-duration acoustic stimulation might be particularly effective in recruiting these local circuits in the IC to produce LSA.

Mostly likely, LSA is a response property that emerges in the IC. Most neurons in the subcortical auditory pathway show a brief suppression of activity after a long-lasting acoustic stimulus^45^,^46^,^47^. However, the dorsal cochlear nucleus (DCN), does show a rebound afterdischarge after a brief period of post-stimulus depression in unanesthetized animals^48^. This afterdischarge may not continue beyond several hundred milliseconds, although it has not likely been examined with the long-lasting stimuli used here. The DCN buildup response does increase during the stimulus. This is shown as facilitation to the second of two successive 25 ms tones and with longer stimuli^41^. It is unclear whether a gradually increasing DCN response during the stimulus would result in a longer afterdischarge lasting into the range seen with LSA. Thus, it is unlikely that LSA in IC neurons is the result of a long-lasting excitatory input from the ascending auditory pathway. On the other hand, a suppressive aftereffect in the many inhibitory pathways that ascend to the IC might contribute to LSA by disinhibition.

Although LSA has not been described previously in the auditory brainstem, the anatomical location of the neurons suggests that this response property could be related to some IC inputs more than others. Most LSA neurons (75%) were in area 1 of ICC. The inputs to ICC are organized into functional zones that receive different subsets of ascending inputs^49^,^50^, and the area corresponding to area 1 in the gerbil receives its most prevalent inputs from the superior olivary complex^51^. However, only 20% of IC neurons had LSA, they were distributed sparsely, and they were likely surrounded by neurons without LSA. This suggests that both LSA and non-LSA neurons may share to same inputs, and makes it less probable that LSA is due to inputs from a single brainstem nucleus.

It is questionable that LSA was due to damage of the periphery. This is unlikely since the stimuli to do so are usually at higher sound levels and longer duration (e.g. 100 dB, 2 hr^52^) than those used here. Even a one week exposure to continuous 80 dB sound produced little change in the auditory threshold in the rat^53^. In fact, a daily 6 hr exposure to 85 dB sound may be protective against traumatic noise exposure and may enhance the cochlea sensitivity^54^. These studies support the notion that LSA was not due to insult of the periphery. However, we cannot rule out a plastic change in the periphery or in the brainstem caused by our sound stimuli.

At this point, we can only speculate on whether the LSA neural behavior results in a percept in the normal hearing animal. Auditory perception is assumed to be directly related to activity in the auditory cortex, but we do not know whether cortical activity is stimulated by IC neurons during the LSA response. The present results show that both GABAergic and glutamatergic IC neurons can exhibit LSA. If the IC neurons with LSA include both the glutamatergic and GABAergic tectothalamic neurons, they would both project to the medial geniculate body that, in turn, supplies the ascending input to auditory cortex. The firing rate of LSA is far higher than the normal spontaneous rate, and those higher rates are similar to those evoked by sound stimulation. Thus, if the tectothalamic pathway is driven by LSA, the auditory forebrain might misinterpret the LSA firing as sound-evoked firing. LSA may not have to be perceived to be useful. The LSA signal may be useful to stimulate the descending auditory pathways in response to prolonged sound stimulation.

Some sound stimuli can evoke auditory afterimages. One example is the Zwicker tone that is a pure tone-like auditory afterimage induced by a noise with a spectral gap or a low pass noise^55^,^56^,^57^,^58^,^59^. The frequency of the Zwicker tone is within the spectral gap or at a frequency above the low-frequency stimulus that produced it. This differs from stimulation at CF with narrow-band noise to induce LSA. The duration of the Zwicker tone is only seconds in most studies with the longest reported being 10 s induced by a 1 min noise^55^. In contrast, the time courses of LSA were diverse (Fig. 2i) and ranged from 17–32 s on average in glutamatergic and GABAergic neurons, respectively, but they could extend to several minutes. Despite these differences, the Zwicker tone and LSA have some properties in common. Both LSA and Zwicker tone became longer when the inducer sound was longer, and both appear to be central in origin and unrelated to changes in the peripheral auditory pathways^58^,^60^.

Another example of an afterimage is the temporary tinnitus that accompanies a temporary threshold shift induced by a narrow band noise at a high sound level (110–120 dB)^61^,^62^. Like LSA, temporary tinnitus is induced in normal hearing individuals and persists for up to 15 minutes after exposure to tone or noise^61^. Unlike LSA, but similar to the Zwicker tone, temporary tinnitus is seldom at the same frequency as the acoustic stimulation and hearing loss^61^,^62^. This suggests that within minutes some type of plastic change may take place in the central auditory pathway in addition to temporary hair cell damage in the cochlea.

LSA might play a role in chronic tinnitus. Chronic tinnitus induced by noise exposure is known to cause long-term plastic change in the auditory pathway^63^. In animal models of chronic tinnitus, hyperactivity in the DCN is well established^64^,^65^,^66^, and it may be caused by a reduction of potassium channel activity^67^ and reduction in GABAergic inhibition^68^. Since the DCN is a major input to the central nucleus of the IC^69^,^70^, it supplies the ICC with a prolonged hyperactive, frequency-specific input after a noise-induced hearing loss, and there is a concurrent increase in spontaneous activity in the IC^71^,^72^. Such hyperactive input might induce LSA in some postsynaptic IC neurons, or it might induce LSA when frequency-specific hyperactivity is paired with sounds lower in frequency than the hyperactive, much like the low-pass noise that produces a higher-frequency Zwicker tone. Indeed, subjects with tinnitus are more likely to hear a Zwicker tone^73^. Thus, a prolonged LSA response in IC neurons induced by a mix of hyperactivity and sound stimulation might contribute to the perception of a phantom sound in tinnitus.

## Methods

### Ethical approval

All experiments were approved by the Animal Care and Use Committee at the University of Connecticut Health Center and done in accordance with institutional guidelines and with the NIH Guide for the Care and Use of Laboratory Animals. All efforts were made to minimize the number of animals used and their suffering.

### Animals

We used thirty transgenic mice expressing channelrhodopsin (VGAT-mhChR2-YFP, Tg(Slc32a1- COP4*H134R/EYFP)8Gfng/J; #14548, Jackson Labs) of either sex (Postnatal day 1.5–4 months). A colony of transgenic mice backcrossed against a C57B/6 background was established. Breeding pairs consisted of one hemizygous and one wild-type or opposite sexed hemizygous. Transgenic offspring had a high expression of EYFP in their brains, and neonates (P0 - 2) were phenotyped by visible fluorescence in the brain under blue light. In additional experiments, we used six female CBA/J (#656, Jackson Labs) mice.

### Sound system

Acoustic stimuli were generated by a TDT System 3 (TDT, Tucker Davis Technologies, Alachua, FL) under the control of custom software (Brian Bishop, UCHC) written in MATLAB (Mathworks, Portola Valley, CA). All sounds were delivered by a closed system that included electrostatic speakers (TDT EC1) coupled to small metal tubes inserted into the external auditory meatus. Binaural acoustic crosstalk was minimal^74^. The sound system was calibrated from 100–100000 Hz. The calibration was performed at the end of the metal tubes with a 1/4″ microphone (Type 4135, Brüel & Kjaer, Naerum, Denmark).

### Surgical Preparation

VGAT-ChR2(H134R)-EYFP mice were anesthetized with a mixture of ketamine (100 mg/kg), xylazine (20 mg/kg) and acepromazine (10 mg/kg), and maintained in an areflexive state with isoflurane (0.5–1%) mixed with oxygen during the surgery and recording. CBA/J mice were anesthetized with urethane (0.9–1.3 g/kg, Sigma). Body temperature was monitored and maintained at >35 °C by a DC temperature controller (FHC, Bowdoin, ME). Vital signs also were monitored (MouseOx Plus, Starr Life Science Corp, PA), and the surgery and recordings were done in a double-walled sound attenuating chamber (IAC, Bronx, NY).

The surgical procedure was described previously^17^,^74^. After the craniotomy, the auditory brainstem response (ABR) to a click (0.5 ms) was measured to verify normal hearing. The threshold of the ABR was around 30 dB SPL (VGAT-ChR2, left, 32.7 ± 1.3 dB, right, 32.3 ± 1.1 dB; CBA/J, left, 31.7 ± 3.1 dB, right, 31.7 ± 1.7 dB). Mice were used for experiments only when the ABR threshold was less than 40 dB.

### Electrophysiology

Single cell extracellular recordings were obtained using glass pipettes filled with 0.01 M PBS (pH 7.4) with 2% Neurobiotin (4–7 MΩ). Glass pipettes were made from borosilicate glass capillaries (34502-99, Kimble Chase, Vineland, NJ). The signals were amplified, bandpass filtered from 300 to 4000 Hz and sampled at 10 kHz with a Multiclamp 700B Amplifier, Digidata 1440A digitizer and Clampex 10.2 system (Molecular Devices). The voltage signals were recorded in current clamp mode. In parallel with recording the signals, the spike times were extracted using a window discriminator and recorded with the TDT System 3 and MATLAB software.

### Optogenetic identification of GABAergic and nonGABAergic neurons

After the single unit was isolated in the VGAT-ChR2 mice, we identified the neuronal type optogenetically (Fig. 1). Light was generated by a blue laser (MBL-ΙΙΙ-473 nm-200 mW, CNI, China) and delivered through an optical fiber (400 μm). The fiber tip was placed several millimeters above the brain surface using a micromanipulator. The light stimulus was a 30 ms light pulse (10–50 mW/mm^2^ at the fiber tip). Light pulses were given every four seconds. Light evoked firing in GABAergic neurons and suppressed spikes in other neurons. We judged that the neuron was suppressed when the light reduced sound evoked spikes by more than 50%. Five neurons with spike suppression by light were juxtacellularly stained and immunohistochemistry confirmed they were not GABAergic and did not contain GAD67 (Fig. 1e).

### Acoustic stimuli

After the cell type identification, the neuron’s best frequency (BF) was determined by 100 ms tone bursts at 70–80 dB. The characteristic frequency (CF) and threshold were also determined by reducing the sound intensity (5 dB step). BF was defined as the frequency where the neuron showed the strongest response. CF was defined as the frequency where the lowest sound level could evoke the response. After determining the BF and CF, we asked whether the neuron had an afterdischarge. In most neurons, we used a 60 dB, 1 octave noise centered at the CF that was either continuous (30–120 s) or discontinuous sound (1 s every 2 s repeated 50 times, or 5 s every 6 s repeated 20 times). We first examined 60 dB sounds and then repeated the stimulation at a higher and/or lower intensity level. In some neurons, we also examined LSA using amplitude modulated (AM) sound with either a sinusoid or a raised sine modulation envelope^75^. We first determined the best modulation frequency using AM one-octave noise (1 s every 2 s) with modulations 2–512 Hz (1 octave steps). Next, LSA was examined by using 30–120 s AM sound at the best modulation frequency. In all LSA experiments, sounds were delivered at least 20 s after the afterdischarge activity returned to the baseline level.

### Data analysis

Data were analyzed with Clampex 10.2 and MATLAB. Spike times were extracted off-line using a fixed threshold. The threshold was set at >5 standard deviations above the baseline. After extracting the spike times, we constructed peristimulus time histograms (PSTH) with a bin size of 500 ms. The PSTH was low-pass filtered (Boxcar, 3 points) to measure the temporal character of the response during sound (RDS) and LSA. To evaluate the duration of the RDS, we measured the width between 25% rise and decay points of the filtered PSTH. When the 25% decay point was not in the RDS, we measured the width between the 25% rise point and sound termination point. For LSA, we measured the peak and decay time. To measure the peak point, we first measured the preceding drop (dip) of the response after sound termination (Fig. 2a,c), and detected the earliest peak point after the dip (Fig. 2a,c). The peak time was the interval between the sound termination and peak point. The LSA decay time was measured as the interval between 10 and 90% points in the decay phase. To evaluate the size of RDS and LSA, we counted the number of spikes in each. The number of RDS spikes was measured as the number of spikes between the sound start and termination, while that of LSA spikes was measured as the number of spikes between the sound termination and LSA termination. The LSA termination was measured as the point where the spike rate returned to the baseline level. The RDS firing rate (Fig. 2b,d) was measured by dividing the number of RDS spikes by sound duration. We measured an accommodation index (AI) to compare the number of spikes in response to the last two seconds of the sound vs. the number of spikes during the first two seconds. For this measurement, we only used the RDS to unmodulated sound. To analyze the responses to discontinuous sound (Fig. 4), we constructed the unfiltered PSTH with a bin size of 1 s. The RDS and the interstimulus responses were measured and compared (Fig. 4b,c).

### Histology

After the single cell recording, the recording site was marked with Neurobiotin by current injection (200 nA, 50% duty cycle of 500 ms, 5 min). In five light suppressed neurons, we performed juxtacellular labeling after the cell type identification, and the recorded neuron was stained by current injection (1–10 nA, 50% duty cycle of 500 ms, 5–20 min). After completion of the electrophysiological recording, animals were given additional anesthesia (ketamine/Xylazine/Acepromazine, 200 mg/kg + 40 mg/kg + 20 mg/kg) and were perfused transcardially and fixed.

The detailed procedure of tissue preparation, immunohistochemistry and imaging was described previously^14^. Briefly, brains were cut at 40 μm, and all primary antibody incubations were overnight. To show the co-localization of ChR2-YFP with GABA, we used rat anti-GFP (1:200, Nacalai Tesque Inc 04404-26, Kyoto, Japan), mouse anti-GAD67 (Millipore MAB5406, 1:3000) and chicken anti-MAP2 (Millipore AB5543, 1:250) for primary antibodies. Secondary antibodies were biotin goat anti-rat (1:200) followed by Streptavidin AF488 (1 mg/ml), AF568 goat anti-MS (1:200), and AF647 goat anti-CKN (1:200). To identify the recording sites, sections were reacted with primary antibodies for GAD67 (Mouse anti-GAD67, Millipore MAB5406, 1:3000) and GLYT2 (Guinea pig anti-GLYT2 Millipore AB1773, 1:10000). After washing, the sections were reacted with secondary antibodies and streptavidin (Invitrogen Streptavidin AF568, 1 mg/mL). Secondary antibodies were AF488 goat anti-Ms (1:200) and AF647 goat anti-GP (1:200). The recording sites were identified by the location of the Neurobiotin-streptavidin labeling in the sections. For juxtacellular labeling, the sections were incubated with antisera to GAD67 (Mouse anti-GAD67, Millipore MAB5406, 1:3000) and VGlut2 (rabbit anti-VGlut2, Synaptic Systems 135 402, 1:1000). After washing, the sections were reacted with secondary antibodies and streptavidin (Invitrogen Streptavidin AF568, 1 mg/mL). Secondary antisera were AF488 goat anti-Ms (1:200) and AF647 goat anti-Rb (1:200). After staining, the sections were mounted, cover slipped and imaged with a Zeiss Axiovert 200M microscope (Carl Zeiss).

### Statistical analysis

All the data are given as mean ± standard error of the mean. For some data, we calculated the correlation coefficient (r), which was statistically tested by the two-tailed Student’s *t* test. When two parameters had an r with significance and r was >0.5, we performed a regression test and plotted a regression line. CFs and thresholds were tested by Kruskal-Wallis test. All other statistical analyses used the Wilcoxon signed-rank test. Criteria for significance were defined as *P* < 0.05.

## Additional Information

**How to cite this article**: Ono, M. *et al.* Long-Lasting Sound-Evoked Afterdischarge in the Auditory Midbrain. *Sci. Rep.* **6**, 20757; doi: 10.1038/srep20757 (2016).

## Figures and Tables

**Table 1:** Mean characteristic frequency and threshold of responses to sound in neurons with and without LSA. Intensity levels required to evoke LSA were ~20 dB above these thresholds.

	n	Characteristic Frequency	Threshold
Neurons With LSA			
GABA	6	20.8 ± 2.2 kHz (11–25 kHz)	31.7 ± 6.4 dB
NonGABA	8	22.8 ± 1.8 kHz (17.5–32 kHz)	39.4 ± 5.8 dB
Neurons Without LSA			
GABA	30	18.5 ± 1.2 kHz, (6.5–38 kHz)	34.7 ± 2.5 dB
NonGABA	32	22.1 ± 1.5 kHz, (5.5–35 kHz)	41.0 ± 2.8 dB
